# Anti-Inflammatory and Phytochemical Analysis of the Crude Leaves Extracts of *Boscia Coriacea Graells* and *Uvaria Leptocladon Oliv*

**DOI:** 10.4314/ejhs.v32i4.19

**Published:** 2022-07

**Authors:** Sintayehu Tsegaye Tseha, Yalemtsehay Mekonnen, Asnake Desalegn, Amelework Eyado, Melaku Wondafarsh

**Affiliations:** 1 Department of Microbial, Cellular and Molecular Biology, College of Natural and Computational Sciences, Addis Ababa University, Ethiopia; 2 Department of Biology, College of Natural and Computational Sciences, Arba Minch University, Ethiopia; 3 Department of Zoological Sciences, College of Natural and Computational Sciences, Addis Ababa University, Ethiopia; 4 Department of Microbial, Cellular and Molecular Biology, College of Natural and Computational Sciences, Addis Ababa University, Ethiopia; 5 Department of Zoological Sciences, College of Natural and Computational Sciences, Addis Ababa University, Addis Ababa, Ethiopia; 6 Department of Plant Biology and Biodiversity Management, College of Natural and Computational Sciences, Addis Ababa University, Ethiopia

**Keywords:** Boscia coriacea Graells, Uvaria leptocladon Oliv, anti-inflammatory activities, phytochemicals

## Abstract

**Background:**

The objective of this study was to evaluate the anti-inflammatory activities and phytochemical composition of the leaves extracts of Boscia coriacea Graells and Uvaria leptocladon Oliv.

**Methods:**

The powdered leaves of Boscia coriacea Graells and Uvaria leptocladon Oliv were extracted by maceration and soxhlet extraction methods. Anti-inflammatory activity of the leaves extracts of Boscia coriacea Graells and Uvaria leptocladon Oliv were evaluated using carrageenan-induced paw edema model. Standard methods were used for analysis of phytochemical composition of the leaves extracts of Boscia coriacea Graells and Uvaria leptocladon Oliv. Data analysis was done using one way analysis of variance.

**Results:**

U. leptocladon Oliv (200 mg/kg) and B. coriacea Graells (200 mg/kg) showed percent inhibition on mice paw edema of 86% and 75% after six hours of carrageenan injection, respectively. The ethanol fraction (100 mg/kg) of U. leptocladon Oliv showed the highest anti-inflammatory effect after six hours of carrageenan injection. The phytochemical analysis of the leaves extracts of B. coriacea and U. leptocladon revealed the presence of tannins, alkaloids, cardiac glycoside, flavonoids, phenols, quinones, and saponins.

**Conclusion:**

The crude leaves extracts of B. coriacea Graells and U. leptocladon Oliv contain phytochemicals with anti-inflammatory activities.

## Introduction

Inflammation is one of the mechanisms of immune response that destroys infectious agents. However, uncontrolled inflammatory response may cause chronic diseases, including diabetes mellitus, cancer, arteritis, cardiovascular and vascular diseases (1). Prolonged use of steroidal anti-inflammatory drugs (SAIDs) and non-steroidal anti-inflammatory drugs (NSAIDs) that are used for the treatment of inflammatory conditions have been shown to cause numerous adverse effects including gastrointestinal bleeding; suppression of immune response; cardiovascular and renal risks (2,3). Therefore, it is necessary to search for safe and effective anti-inflammatory drugs.

Medicinal plants are major sources of chemicals with anti-inflammatory activities. Some medicinal plants that belong to the genus *Uvaria* and *Boscia* have anti-inflammatory properties (4,5). The genus *Boscia* belongs to the family Capparidaceae (6), which is widely distributed in Ethiopia. It is found in Bale, Sidamo, Kefa, Konso, Gamo Gofa and Hararge regions of Ethiopia (7). The genus *Uvaria* belongs to the family Annonaceae that is also widely distributed in Ethiopia. For example, *Uvaria leptocladon* is found in Sidamo, Kefa, Konso (Alie), and Gamo Gofa regions of Ethiopia (8). Thus, the objective of this study was to evaluate the anti-inflammatory activities and phytochemical composition of the crude leaves extracts of *B. coriacea Graells* and *U. leptocladon Oliv.*

## Materials and Methods

**Plant collection**: Fresh leaves of *B. coriacea Graells* and *U. leptocladon Oliv* were collected in April 2021 from Alie and Konso, which are located in Southern Ethiopia. After the plant materials were authenticated by one of the authors, a Botanist at the Department of Plant Biology and Biodiversity Management, a voucher specimen of each plant (ST001 and ST002, representing *B. coriacea Graells* and *U. leptocladon Oliv*, respectively) was deposited at the National Herbarium, College of Natural and Computational Sciences, Addis Ababa University. Then, the leaves samples were cleaned and allowed to dry in shade a voiding direct sun light.

**Extraction**: The powdered leaves of *B. coriacea Graells* (100 g) and *U. leptocladon Oliv* (100 g) were extracted by soxhlet extraction method using a liter (1 L) of 80% methanol (MeOH). In addition to this, the powdered leaves of *B. coriacea Graells* (1 kg) and *U. leptocladon Oliv* (1 kg) were extracted by maceration method using 10 liters of 80% MeOH. Then, the mixture was filtered using Whatmann no.1 filter paper. The MeOH was removed from the filtrate by evaporation using rotary evaporator. The water component of the mixture was removed by lyophilization using a lyophilizer equipment (Christ, ALPHA 2-4-LD Plus). The 80% MeOH extract was fractionated using chloroform (CHCl_3_), MeOH and CHCl_3_-MeOH (1:1) in the Department of Chemistry, College of Natural and Computational Sciences, Addis Ababa University. The extraction yields (w/w) were 5% and 18% for maceration and soxhlet extracts, respectively. All the extracts were stored at -20°C until experiments were conducted.

**Evaluation of anti-inflammatory activity**: The anti-inflammatory activities of the leaves extracts of *B. coriacea Graells* and *U. leptocladon Oliv* were evaluated by carrageenan induced paw edema model (9). First, the Swiss Albino mice were randomly grouped into three groups, each group containing six mice. Thirty minutes before carrageenan injection, the first group was given leaf extract and the second and third groups were orally treated with positive control (indomethacin) and negative control (normal saline). After thirty minutes, 50 µl of 1% freshly prepared solution of carrageenan was injected into the left-hind paw of each mouse. For each mouse, the initial diameter of the left hind paw was measured using micrometer (Digimatic micrometer, Mitu Toyo Corporation) before administration of the extracts. The diameter of paw was also measured post-carrageenan injection at time zero (just after carrageenan injection), and every hour for six consecutive hours (1h, 2h, 3h, 4h, 5h and 6h after carrageenan injection).

Percentage reduction of paw edema was calculated using the following formula:-

(Vt-V0 in control group)-(Vt-V0 in treatment group) x100

Vt-V0 in control group

Where,

V0 = is the mean diameter of paw before injection of carrageenan in treated and negative control groups and

Vt= is the mean diameter of paw in treated and negative control groups after carrageenan injection at time t.

**Phytochemical analysis**: The preliminary phytochemical analysis of the 80% methanol leaves extracts of *B. coriacea Graells* and *U. leptocladon Oliv* was done using standard methods (10–13).

**Ethical considerations**: Ethical clearance was obtained from the Institutional Review Board of the College of Natural and Computational Sciences of Addis Ababa University. Copy is attached as additional file.

**Data analysis**: Analysis of differences between anti-inflammatory activities of the fractions of *U. leptocladon Oliv* leaf extract was done by using one way analysis of variance with post hoc comparison (Tukey's test).

## Results

**Anti-inflammatory activity of the leaves extracts of *B. coriacea Graells* and *U. leptocladon Oliv***: The leaves extracts of both *B. coriacea Graells* and *U. leptocladon Oliv* showed anti-inflammatory effects in carrageenan-induced paw edema model. *U. leptocladon Oliv* (200 mg/kg) and *B. coriacea Graells* (200 mg/kg) showed percentage inhibition on mice paw edema of 86% and 75% after six hours of carrageenan injection, respectively (Table 1). As shown in Table 2, the ethanol fraction (100 mg/kg) of the leaf extract of *U. leptocladon Oliv* demonstrated the highest (77.7%) anti-inflammatory effect after six hours of carrageenan injection as compared with ethyl acetate (50%) and chloroform (66.7%) fractions.

**Table 1:** Anti-inflammatory activity of 80% methanol leaves extracts of *B. coriacea Graells* and *U. leptocladon Oliv* (200 mg/kg) using carrageenan-induced paw edema model

Plant extract and positive control	Time after carrageenan injection in hour					

	1^st^ hour	2^nd^ hour	3^rd^ hour	4^th^ hour	5^th^ hour	6^th^ hour
Indomethacin	7.1%	26.7%	46.7%	56.2%	68.7%	87.3%
*U.leptocladon*	7.1%	40%	40%	50%	68.7%	86%
*B.coriacea*	21.4%	40%	46.7%	56.2%	62.5	75%

Note: Indomethacin (25 mg/kg) was used as positive control

**Table 2:** Anti-inflammatory activity of fractions of the leaf extract of *U. leptocladon Oliv* (100 mg/kg) using carrageenan-induced paw edema model

Fractions of *U.* *leptocladon* and positive control	Time after carrageenan injection in hour					

	1^st^ hour	2^nd^ hour	3^rd^ hour	4^th^ hour	5^th^ hour	6^th^ hour
Indomethacin	6.25%	31.2%	50%	52.9%	72.2%	88.9%
Ethanol	25%	25%	37.5%	38.8%	61.1%	77.7%
Chloroform	25%	25%	31.2%	41.2%	44.4%	66.7%
Ethyl-acetate	12.5%	12.5%	12.5%	17.6%	22.2%	50%

Note: Indomethacin (25 mg/kg) was used as positive control

**Phytochemical composition of the leaves extracts of *B. coriacea Graells* and *U. leptocladon Oliv***: The preliminary phytochemical screening of 80% methanol leaves extracts of *B. coriacea Graells* and *U. leptocladon Oliv* revealed the presence of tannins, alkaloids, flavonoids, cardiac glycosides, phenols, quinones, and saponins. In addition to the aforementioned phytochemicals, the leaf extract of *U. leptocladon Oliv* contains terpenoids and steroids (Table 3).

**Table 3:** Phytochemical composition of 80% methanol leaves extracts of *B. coriacea Graells* and *U. leptocladon Oliv*.

Types of secondary metabolites	Types of plant species	

	*U. leptocladon*	*B. coriacea*
Tannins	+	+
Alkaloids	+	+
Flavonoids	+	+
Cardiac glycosides	+	+
Terpenoids	+	-
Phenols	+	+
Quinones	+	+
Saponins	+	+
Steroids	+	-

**Note**: The plus sign (+) indicates the presence of a secondary metabolite in the plant extract, whereas the minus sign (-) indicates the absence of a secondary metabolite in the plant extract

## Discussion

The anti-inflammatory activity of medicinal plants is related to the presence of phytochemicals such as saponins, alkaloids, tannins, cardiac glycosides, and flavonoids (14,15). The anti-inflammatory activities of the crude leaves extracts of *B. coriacea Graells* and *U. leptocladon Oliv* observed in this study might be due to the presence of saponins, alkaloids, cardiac glycosides, flavonoids, and tannins in the leaves extracts. The anti-inflammatory activity of the *U. leptocladon Oliv* observed in this study is comparable with the findings of other studies that used carrageenan-induced paw edema model to assess the anti-inflammatory activity of the leaf extract of *Uvaria chamea* (4,16). The highest anti-inflammatory activity of the ethanol fraction of *U. leptocladon Oliv* leaf extract suggests that the inflammatory constituents are contained in this fraction.

Acute inflammation induced by carrageenan is linked with the induction of production of inflammatory mediators such as histamines, serotonins, bradykinin and prostaglandins (17). Therefore, the inhibition of paw edema in the mice that were administered with the leaves extracts of *B. coriacea Graells* and *U. leptocladon Oliv* could be due to the inhibition of production of serotonin, histamine, bradykinin and prostaglandins by the phytochemicals that are found in the leaves extracts of *B. coriacea Graells* and *U. leptocladon Oliv.*

The phytochemical constituents of the leaves extracts of *U. leptocladon Oliv* and *B. coriacea Graells* identified in this study is also comparable with the findings of other researchers that investigated the phytochemical composition of the leaves extracts of other plants that belongs to the genus *Uvaria* such as *U. chamae* (4,16,18,19) and the genus *Boscia* such as *B. senegalensis, B. arabica* and *B. variabilis* (20,21).

In conclusion, 1. The 80% MeOH leaves extracts of *U. leptocladon Oliv* and *B. coriacea Graells* contain tannins, alkaloids, flavonoids, cardiac glycosides, phenols, quinones, and saponins, 2. The leaves extracts of *U. leptocladon Oliv* and *B. coriacea Graells* have anti-inflammatory activities.
